# Human gut microbiome gene co-expression network reveals a loss in taxonomic and functional diversity in Parkinson’s disease

**DOI:** 10.1038/s41522-025-00780-0

**Published:** 2025-07-24

**Authors:** Rémy Villette, Polina V. Novikova, Cédric C. Laczny, Brit Mollenhauer, Patrick May, Paul Wilmes

**Affiliations:** 1 Luxembourg Centre for Systems Biomedicine, University of Luxembourg, Esch-sur-Alzette, Luxembourg; 2 Department of Neurology, University Medical Center Göttingen, Göttingen, Germany; 3 Paracelsus-Elena-Klinik, Kassel, Germany; 4 Department of Life Sciences and Medicine, Faculty of Science, Technology and Medicine, University of Luxembourg, Esch-sur-Alzette, Luxembourg

**Keywords:** Microbial communities, Microbial genetics

## Abstract

Gut microbiome alterations are linked to various diseases, including neurodegeneration, but their ecological and functional impacts remain unclear. Using integrated multi-omics (metagenomics and metatranscriptomics), we analyse microbiome gene co-expression networks in Parkinson’s disease (PD) and healthy controls (HC). We observe a significant depletion of hub genes in PD, including genes involved in secondary bile acid biosynthesis, bacterial microcompartments (BMCs), polysaccharides transport and flagellar assembly (FA). *Blautia*, *Roseburia*, *Faecalibacterium* and *Anaerobutyricum* genera are the main contributors to these functions, showing significantly lower expression in PD. Additionally, we identify a strong correlation between BMC and FA expression, and an apparent dysregulation in cross-feeding between commensals in PD. Finally, PD also exhibits reduced gene expression diversity compared to HC, whereby higher gene expression correlates with greater diversity. We identify disruptions in gut metabolic functions, at both taxonomic and functional level, and microbiome-wide ecological features, highlighting targets for future gut microbiome restoration efforts.

## Introduction

Parkinson’s disease (PD) is the second most prevalent neurodegenerative disorder, primarily caused by the loss of dopaminergic neurons and the formation of Lewy bodies in the brain as the major pathological hallmarks^1,2^. PD is characterised by both motor and non-motor symptoms, with non-motor symptoms such as dysphagia, constipation, and bloating being linked to the gastrointestinal tract. Notably, idiopathic constipation, a common symptom of PD, often precedes motor symptoms by over a decade^3^, supporting the hypothesis that the disease can originate in the gut^4^. Intestinal dysbiosis has been documented in PD^5–7^, and evidence suggests that the gut might initiate or worsen the development of PD^8–10^. Changes to gut microbiome structure can compromise gut permeability and the integrity of the intestinal barrier, affecting gastrointestinal epithelial cells, the immune system, and the enteric nervous system^11,12^. Moreover, a dysbiotic gut is characterised by decreased microbial diversity^11^. Multiple studies have uncovered evidence for dysbiosis in the gut microbiome of individuals with PD characterised by shifts in bacterial and archaeal taxa including *Akkermansia, Roseburia, Methanobrevibacter*^13–18^. In addition, we recently showed a decreased activity for *Roseburia sp*., *Blautia obeum*/*wexlerae* and *Blautia massiliensis* in PD, a significant decrease in transcripts implicated in flagellar assembly and chemotaxis, and differences in the gut metabolome including decrease levels of chenodeoxycholic, glycocholic acid, glucuronic acid and glycerol, as well as an increase in β-glutamate, isovalerate and isobutyate^19^

To further explore the reasons behind the taxonomical changes reported by the literature and the recent findings we provide, we opted for complementary approaches to the more traditional bioinformatic and biostatistics methods. Indeed, differential expression analysis or multi-variate approaches do not capture the importance of functions within a complex ecosystem such as the gut microbiome. For that purpose, gene co-expression networks have been widely used to uncover functional gene clusters and pathways associated with various disease phenotypes^20,21^. In this approach, co-expressed genes are clustered into modules based on network properties which allows us to investigate their biological properties but also relations between different biological process. In addition to gene clustering, we can also define hub genes based on their importance for the network and for modules (intramodular Hub genes, iHub genes).

Here, we employ weighted gene co-expression network analysis (WGCNA) based on the ratio of metatranscriptomic (MT) to metagenomic reads transcript per million (MG TPM) from the gut microbiomes of individuals with PD and healthy individuals, referred here as healthy controls (HC). Using this approach, we managed to describe important shifts in the bacterial ecosystem metabolism, especially regarding bacterial microcompartments (BMCs), polyols transporters. Finally, we highlight the cross talk between commensal species and the decrease of taxonomic diversity of gene expression (tDGE) in PD compared to HC.

## Results

### Microbial transcriptional activity is linked to disease status

**Fig. 1:**
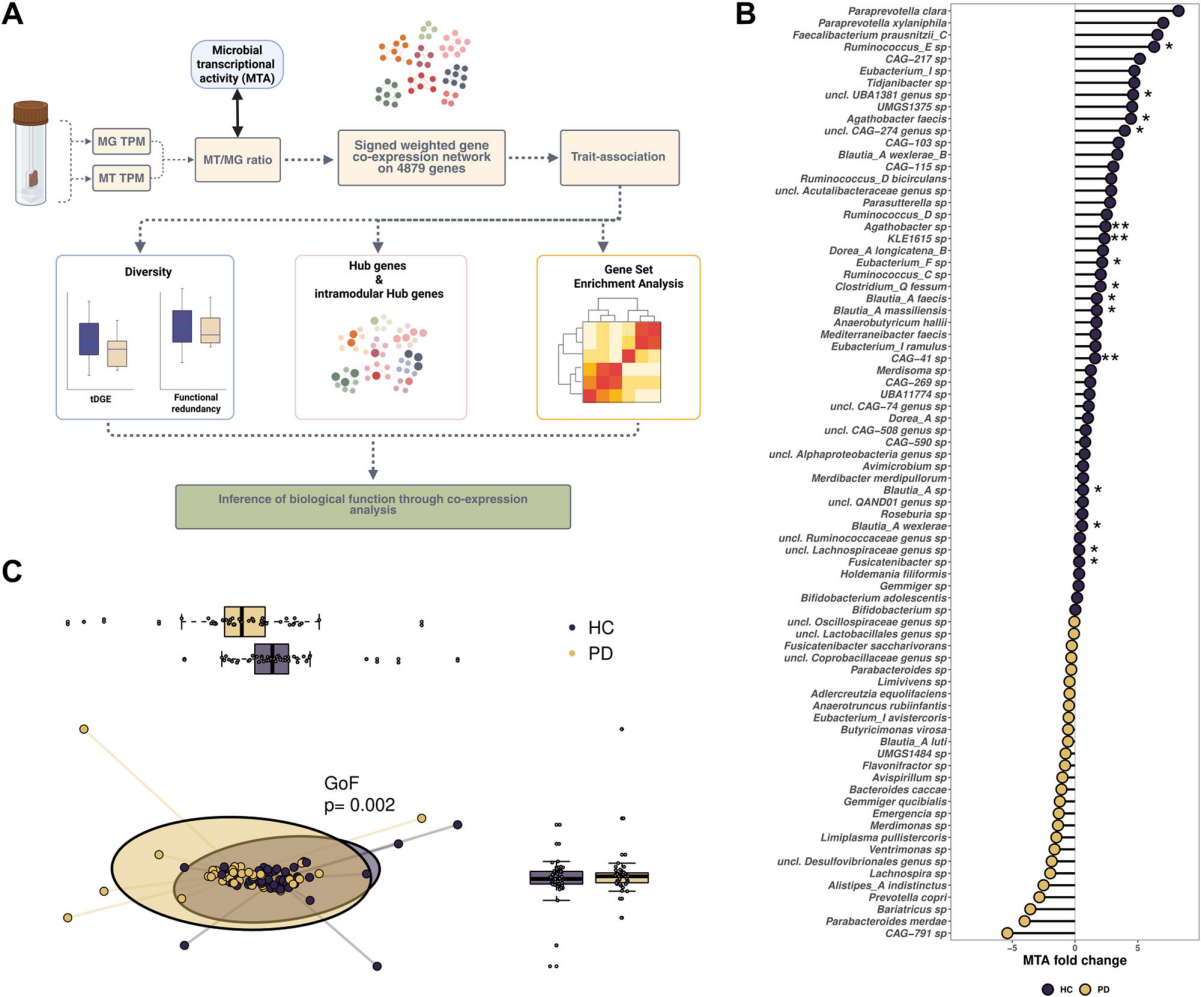
Analysis workflow and analysis of microbial transcriptional activity taxonomically resolved. **A** Metagenomic (MG) and metatranscriptomic (MT) counts per gene were converted into values representing the normalized gene expression MT/MG ratio. A co-expression gene network was then constructed based on a dataset of 4879 genes derived from both PD and HC individuals. This network revealed 17 distinct gene modules. Among these, modules significantly associated with either PD or HC were selected. Further analyses focused on gene diversity analysis, hub genes and gene set enrichments, aiming to uncover the ecological relevance of these modules in relation to the disease. Gene set enrichment analysis (GSEA), Parkinson’s disease (PD), healthy control (HC). **B** Differential MTA analysis at the species level. All taxa represented have been filtered on 50% prevalence in the cohort and a *p* < 0.05. Asterisks represent species differentially active after FDR correction. **C** Principal component analysis of MTA between HC and PD, MTA values have been scaled using Centered Log Ratio. *P* value results from environmental factor fitting coined GoF, for Goodness of fit.

We previously showed metatranscriptomic differences in the gut microbiome of individuals with PD^19^, whereby *Faecalibacterium*, *Roseburia* and *Blautia* had decreased expression levels in PD gut microbiome. In addition, we found a strong decrease of flagellar assembly expression in PD, which led us to investigate the gut microbiome of PD in a network approach to shed light on the gene co-expression patterns that are linked to the differences stated above. For this purpose, we started by using a complementary approach consisting of using an abundance-normalised gene expression data, more specifically the ratio of MT and MG transcripts per million (TPM, Fig. 1A)^22^. We propose here to use this ratio as a proxy of microbial transcriptional activity (MTA) by investigating the differences between gene abundance/potential and gene expression. We used a cohort of 49 healthy controls (HC) and 46 PD donors for which groups were not different in age, sex, diabetes or drug consumption, only constipation was more present in the PD group compared to HC (p < 0.05, Chi-square test, Supplementary Data 1).

We started by assessing the overlap of gene and species in the dataset. We found that that 59% and 62% of the genes were found in more than half of the samples per group (Supplementary Fig. 1A). In addition, we found that around 2500 genes are shared between all individuals while most of the species are sample dependant since most of the species are unique to an individual (Supplementary Fig. 1B, C). Interestingly, we found that genes that are the most prevalent have a high average and median MTA, but also a high variance compared to the less prevalent genes (Supplementary Fig. 1D). In addition, we compared the differences of MTA between HC and PD. This revealed a decrease of MTA in genera such as *Blautia*, *Eubacterium* and *Agathobacter* (*q* < 0.05, Supplementary Fig. 2A). Species level analysis revealed decreased MTA for *Ruminococcus*_E sp., *Fusicatenibacter* sp., *Blautia* species *wexlerae*, *faecis* and *massiliensis*, and *Agathobacter* species *faecis* and sp. (*q* < 0.05, Fig. 1B and Supplementary Fig. 2). Interestingly, we found no significant increase of MTA in PD microbiome at the genera and species level, indicating a loss of transcriptional activity but not an increase (*q* > 0.05, Fig. 1B and Supplementary Fig. 1A, B). Finally, we checked if these differences at the genera and species level were segregating the two groups, for this purpose, we used a multiple reduction approach on centred log ratio of MTA values. We found a significant difference between the two groups using environmental vector fitting (Goodness of fit = 0.02, Fig. 1C).

### Microbial co-expression network is linked to disease status

**Fig. 2:**
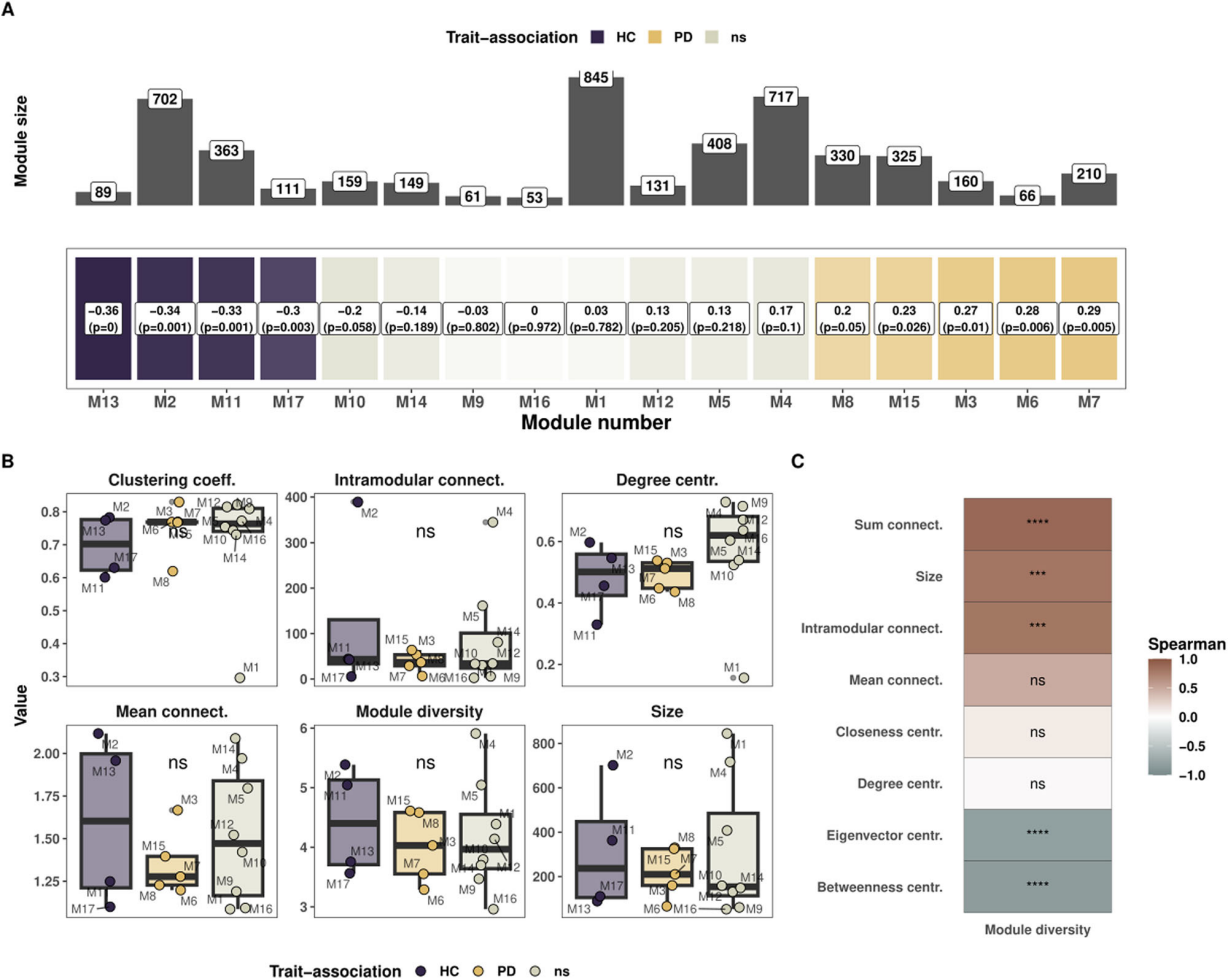
Weighted correlation gene network analysis reveals module associations with disease. **A** Module trait relationship heatmap with correlation and p-values for each module. Modules are sorted from left to right (healthy to Parkinson’s disease) based on the eigenvector value. The top panel represents the number of genes belonging to each module. **B** Network topology analysis for the modules grouped by trait association. A Kruskal and Wallis test was performed according to the trait association to compare modules based on their associations or not to one of the two groups. **C** Correlation between module diversity and other topology features. Correlation tests are spearman tests.

To delve into the inter-relation between microbes in PD gut microbiome we constructed a co-expression network, using the MTA approach described above. For this purpose, we used WGCNA algorithm to capture the gene co-expression patterns^23^. From an original set of 11,876 microbial KOs, referred in this paper as genes, we constructed a signed co-expression network of 4789 genes that were shared by at least 50% of the donors. The co-expression network revealed 17 modules from which trait-association was found, four significantly associated with HC (M13, M2, M11 and M17, *p* < 0.05), five significantly associated with PD (M3, M6, M7, M8 and M15, *p* ≤ 0.05), and eight neither significantly associated with HC nor with PD (M1, M4, M5, M9, M10, M12, M14 and M16, Fig. 2A).

We next looked at network topological features, with the addition of the module diversity, which we define by the diversity of genes found within each module, using the Shannon index^24^. Modules M2 and M4 showed high mean connectivity, highest intramodular connectivity and highest module diversity despite their large size (Fig. 2B and Supplementary Fig. 1). M1 had the lowest clustering coefficient, degree centrality, eigenvector centrality and closeness centrality, thereby demonstrating that M1 acted as a default module, comprised of genes with the lowest clustering coefficient in the dataset (Fig. 2B and Supplementary Fig. 3). M6 exhibited high values of betweenness and eigenvector centrality but low connectivity (Fig. 2B and Supplementary Fig. 3A). When analysing the modules based on trait association, we found no statistically significant differences in topological measures but important variation between the modules, irrespective of trait association (Kruskal and Wallis test, *p* > 0.05, Fig. 2B and Supplementary Fig. 3A). However, M1 being a clear outlier for clustering coefficient, degree and closeness centrality, we removed it from the statistical testing to re-assess modules based on trait association and did indeed find a significantly higher degree and closeness centrality for modules not associated to either to health-disease status (Kruskal and Wallis, *p* = 0.023 and *p* = 0.013, data not shown). Finally, it was apparent that module diversity and the different topology metrics such as the sum of connectivity and intramodular connectivity were correlated (*R*^2^ > 0.8, *p* < 0.001, Fig. 2C). In contrast, eigenvector and betweenness centrality were anticorrelated with module diversity (*R* < -0.8, *p* < 0.001, Fig. 2C). Overall, we found significant anti-correlations between connectivity measures and closeness/betweenness centralities while the clustering coefficient was correlated to degree/eigenvector centrality (Supplementary Fig. 3C).

### Modules associated with healthy individuals exhibit enrichment in flagellar assembly and secondary bile acid biosynthesis

**Fig. 3:**
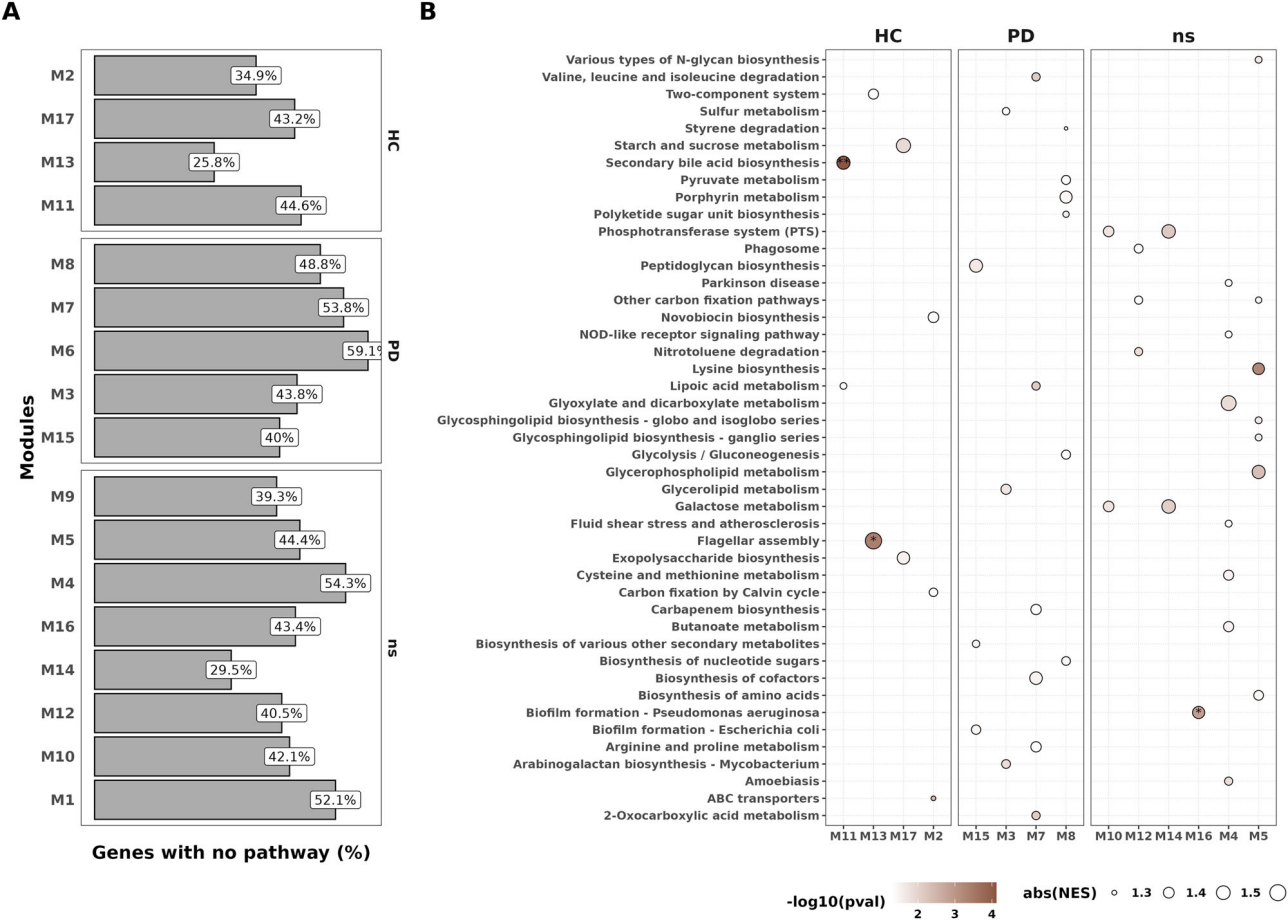
Gene set enrichment analysis on KEGG pathway highlights enrichment in HC modules but not in PD modules. **A**. Count of genes within a module with undescribed pathways or not belonging to any KEGG pathway. **B**. Gene Set Enrichment Analysis of the different modules. All dots plotted are representing a significant enrichment before correction (*p* < 0.05), coloured by −log10(*p* value). Asterisks represent significant enrichments after FDR correction (*q* < 0.05).

To describe biological processes involved in the different modules, we next proceeded to gene set enrichment analysis (GSEA) to resolve the pathway-level information using KEGG pathways. We however faced a striking challenge as we found that on average 43.7% (min: 26%, max: 59%, Fig. 3A) of genes within modules did not belong to any pathway, some genes also having no described function. With respect to known pathways and function, modules were comprised on average of 28.5 different pathways ranging from 10 (M6) to 42 pathways (M1) (Supplementary Fig. 4). We identified an enrichment in flagellar assembly in M13 and secondary bile acid biosynthesis in M11, which, in both cases, were associated with HC (*q* < 0.05, Fig. 3B). We also found an enrichment in genes involved in biofilm formation for M16 (no trait association) and no statistically significant enrichment in PD-associated modules (Fig. 3, *q* < 0.05, *q* < 0.05 and *q* > 0.05, respectively, GSEA). Although not statistically significant after correction, we noticed enrichments in the following pathways within modules associated with PD: glycerolipid metabolism (M3), peptidoglycan biosynthesis (M15), lipoic acid metabolism and valine degradation (M7) (Fig. 3B, *p* < 0.01, *q* > 0.05). We also noticed the presence of beta-lactam resistance genes (*oppA, oppB, oppC, oppD* and *oppF*) and quorum sensing genes in PD-associated module M6 (Supplementary Fig. 4). In addition, genes involved in methane metabolism were present in the PD-associated modules M6 and M8 (Supplementary Fig. 4). GSEA results can be found in Supplementary Data 2.

### Hub genes are restricted to health-associated modules

**Fig. 4:**
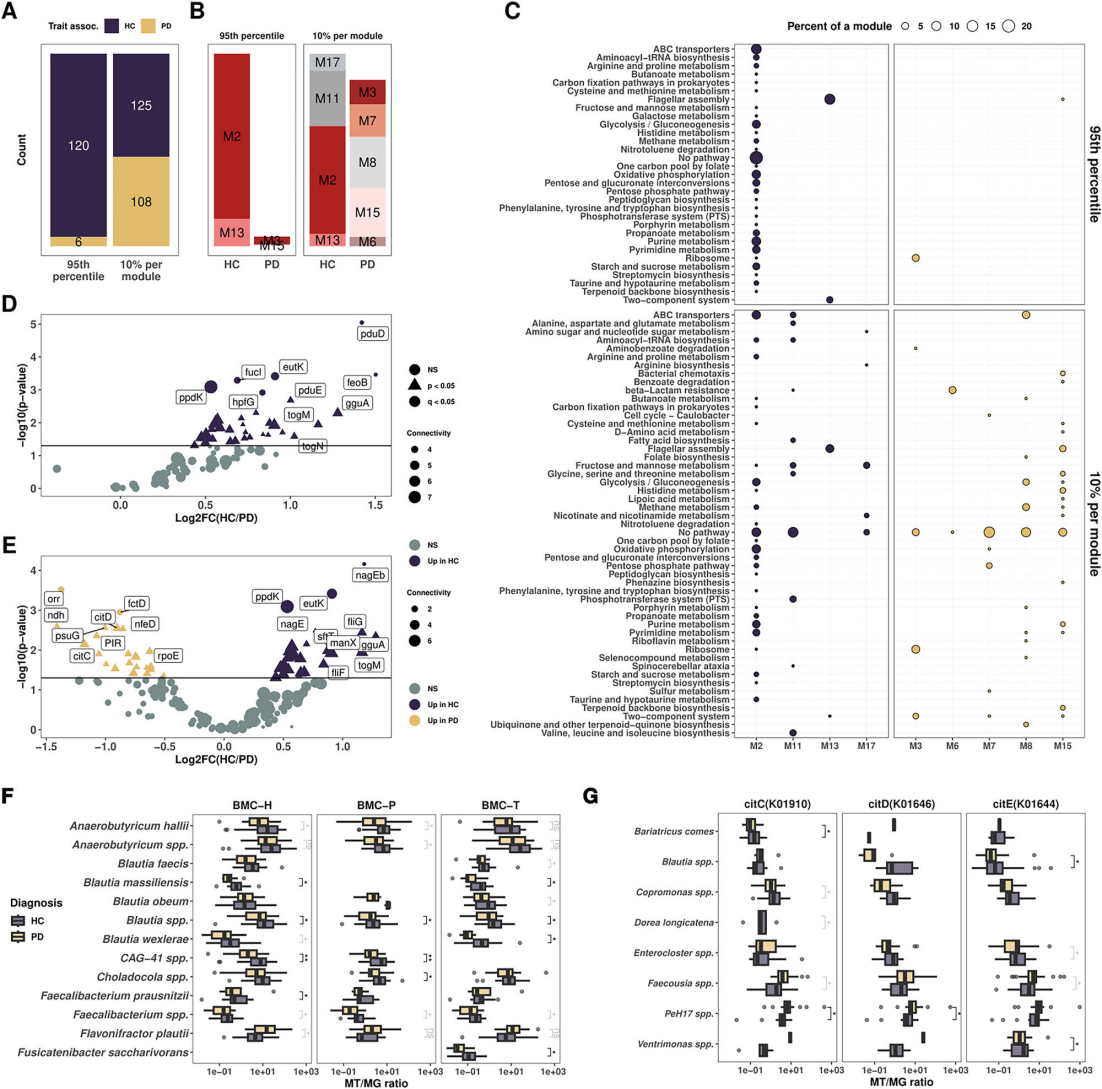
Hub genes are mostly associated with healthy individuals. **A** Bar plot highlighting the counts of hub genes selected based on top 100 connected genes (**A**) and 10% top connected genes per module (**B**). **C** Dot plot representing the count of pathways per module for the hub genes and iHub genes. The size of the dots represents the proportion of a given pathway within a module. **D**, **E** Volcano plots of differential expression of genes for the hub genes selected with the 95th percentile connected genes in the network (**D**) and top 10% connected per modules, also called iHub genes (**E**). Dots are colorized by group and shaped on the level of significance, triangular shape for *p* < 0.05 and round coloured shape for *q* < 0.05. **F**, **G**. Boxplots representing gene normalized expression resolved at the species level for the BMC shell proteins (**F**) and citrate lyases (**G**). All tests are Wilcoxon signed rank test with *q* values (FDR corrected) depicted in black and *p* values (*q* > 0.05 after FDR correction) depicted in grey.

Complementary to GSEA analysis, we next looked at hub genes to assess key functions within the microbial co-expression network, enabling us to capture the most important genes in this network and give more understanding on the module’s biological roles. We first selected the 95th percentile of genes to select the top 5% of genes with the highest connectivity from all modules associated with HC and PD, excluding genes from modules not associated with either of the groups. Out of the 126 genes identified as 95th percentile, 120 were from HC-associated modules and 6 from PD-associated modules (Fig. 4A). To resolve more genes from PD-associated modules, we selected 10% of the most connected genes within each module, representing here intramodular hub genes (iHub genes), thereby retrieving 125 from HC-associated modules and 108 from PD-associated modules (Fig. 4A). For the 95th percentile approach, most genes belonged to module M2 (82%) and the rest belonged to M13 (13.5%), M3 (3.9%) and M15 (0.8%, Fig. 4B). Hub genes associated with HC were mainly involved in energy production (oxidative phosphorylation, glycolysis/gluconeogenesis), transporter activity (ABC transporters), nucleotide metabolism (pyrimidine and purine metabolism), saccharide metabolism (pentose and glucuronate interconversions, starch and sucrose metabolism) as well as microbial motility (flagellar assembly and two-component system) (Fig. 4C). With the 10% per module approach, we noted the presence of two glutamate synthases (*GLT1* and *gltB*) in the module M11 linked to alanine, aspartate and glutamate metabolism (Fig. 4C).

Amongst the hub genes, we found a significant increase in their expression in HC including flagellar assembly (*flgB, fliQ, fliS, flgE, fliK, flgL, fliD, fliP*, *p* < 0.05, Fig. 4D, Supplementary Data 3). We noted that genes involved in bacterial BMC catabolism and metabolism (eut and pdu operons) were significantly increased in HC (*eutL, pduE, eutK*, *q* < 0.05, DeSeq2, Fig. 4D, E) along with genes involved in polyols transport (togB, togM, togN, gguA, lacF and ganA, *p* < 0.05, *q* > 0.05, DeSeq2 Fig. 4D, E). Of note, the togBMNA operon is an important transporter for oligogalacturonide, a by-product of pectin degradation and involved in short chain fatty acid (SCFA) production. We also noted a significantly increased expression for citrate lyase genes in individuals with PD (*citD, citC* and *citE*, *p* < 0.05, *q* > 0.05, Fig. 4E).

We then proceed to taxonomically resolve the expression of these genes. First, we noted the variability in ortholog annotations between the pdu and eut operons, these annotations were dependent on the taxonomy. Indeed, most of the shell proteins annotated as members of the eut operon, while catabolism/anabolism genes were annotated as members of the pdu operon. We manually grouped the genes according to their described functions in the literature. Resolving the gene expression at the species level revealed a significant decrease in expression for these genes in PD, including in *Blautia* spp.*, Blautia massiliensis, Blautia obeum*, *Blautia massiliensis, CAG-41 spp., Anaerobutyricum soehngenii* and *Faecalibacterium prausnitzii* (*q* < 0.05, Wilcoxon test, Fig. 4F and Supplementary Fig. 5A). Of note, this decrease was also observed at the genus level (data not shown). Interestingly, we found an increase in *Flavonifractor plautii*’s expression of BMC genes encoding for BMC-H and pduQ in PD (*p* < 0.05, Wilcoxon test, Supplementary Fig. 5A). In addition, we noted a decrease of expression in for togBMN genes in *F. prausnitzii* and *Gemmiger qucibialis*, for lacFG, gguA and wzm in *Blautia spp*., and for ganPQ and msmX in *Agathobacter spp*. (*q* < 0.05, Wilcoxon test, Supplementary Fig. 5B-C). Finally, citrate lyase genes expression revealed the increased expression of *PeH17 spp*. and *Faecousia spp*. in individuals with PD (*q* < 0.05 and *p* < 0.02, respectively, Wilcoxon test, Fig. 4G).

### Bacterial microcompartment genes are correlated with flagellar expression

**Fig. 5:**
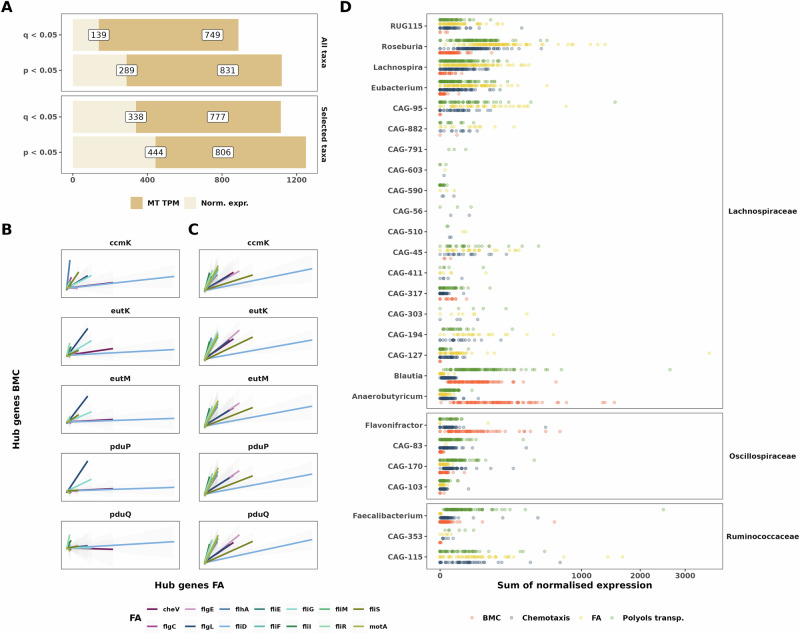
Bacterial microcompartments are correlated with genes involved in flagellar assembly. **A** Bar plots highlighting the number of positive correlations before and after FDR correction for normalized expression and MT TPM when taking all taxa expressing the BMCs genes and FA genes (upper panel) and relevant taxa from Fig. 4 (lower panel). All tests are based on the Spearman correlation. Correlation plots including selected taxa, both for normalized expression (**B**) and MT TPM (**C**), considering only hub genes from the 10% per modules approach. Tests are based on the Spearman correlation and all correlations are significant after FDR correction (*q* < 0.05). **D** Dot plot representing the sum of normalised expression per sample for a given process. BMC, FA and Chemotaxis gene grouping comprise all genes detected in the dataset, while ABC transporters only include the following genes: togBMNA, lacDEFGAICR, man, gguAB, ganAQ, msmEFGX, and wzm. Genera represented here are the “selected taxa” presented in (**A**, **B**).

We next investigated the links between the expression of BMC genes and FA genes. For this, we tested the correlation between these genes using both normalised gene expression (reminder: MT/MG ratio) and MT TPM. In addition, we tested the correlations for all the genes from all the taxa or selected taxa based on literature evidence and evidence in this work (see ‘Materials and methods’ for details). We found strong correlations between the levels of gene expression for BMC and FA genes (Fig. 5A and Supplementary Fig. 6A-B). Indeed, when using all the taxa from the dataset we noted 749 significant correlations before FDR correction and 139 significant correlations after FDR correction when using normalised gene expression (Fig. 5A, *p* < 0.05, *q* < 0.05, respectively, Spearman correlation test); 831 significant correlations before FDR and 289 significant correlations after FDR correction when using MT TPM (Fig. 5A, *p* < 0.05, *q* < 0.05, respectively, Spearman correlation test). In addition, using only the selected taxa resulted in even more significant correlations both for normalized gene expression (444 genes with *p* < 0.05, 338 genes with *q* < 0.05, respectively, Spearman correlation test) and MT TPM (806 genes with *p* < 0.05, 777 genes *q* < 0.05, respectively, Fig. 5A). Next, we specifically checked the correlation of hub genes for the selected taxa, and we found significant correlations between hub genes from BMCs and FA genes both for normalized expression and MT TPM (Fig. 5B, C, *q* < 0.05, Spearman correlation test). Finally, we investigated the shared presence of the different processes in the identified taxa. Interestingly, we found that *Blautia*, *Anaerobutyricum* and *Flavonifractor* were expressing mainly ABC transporters and BMC genes while *Roseburia*, *Lachnospira* and *Eubacterium* expressed almost no BMC genes (Fig. 5D). We also noted that many cryptic genera, such as CAG-115, did not express BMC genes (Fig. 5D). The correlations between BMC and FA genes are highlighting a cross-feeding process between the commensals.

### Genes enriched in Parkinson’s disease exhibit lower taxonomic diversity of gene expression

**Fig. 6:**
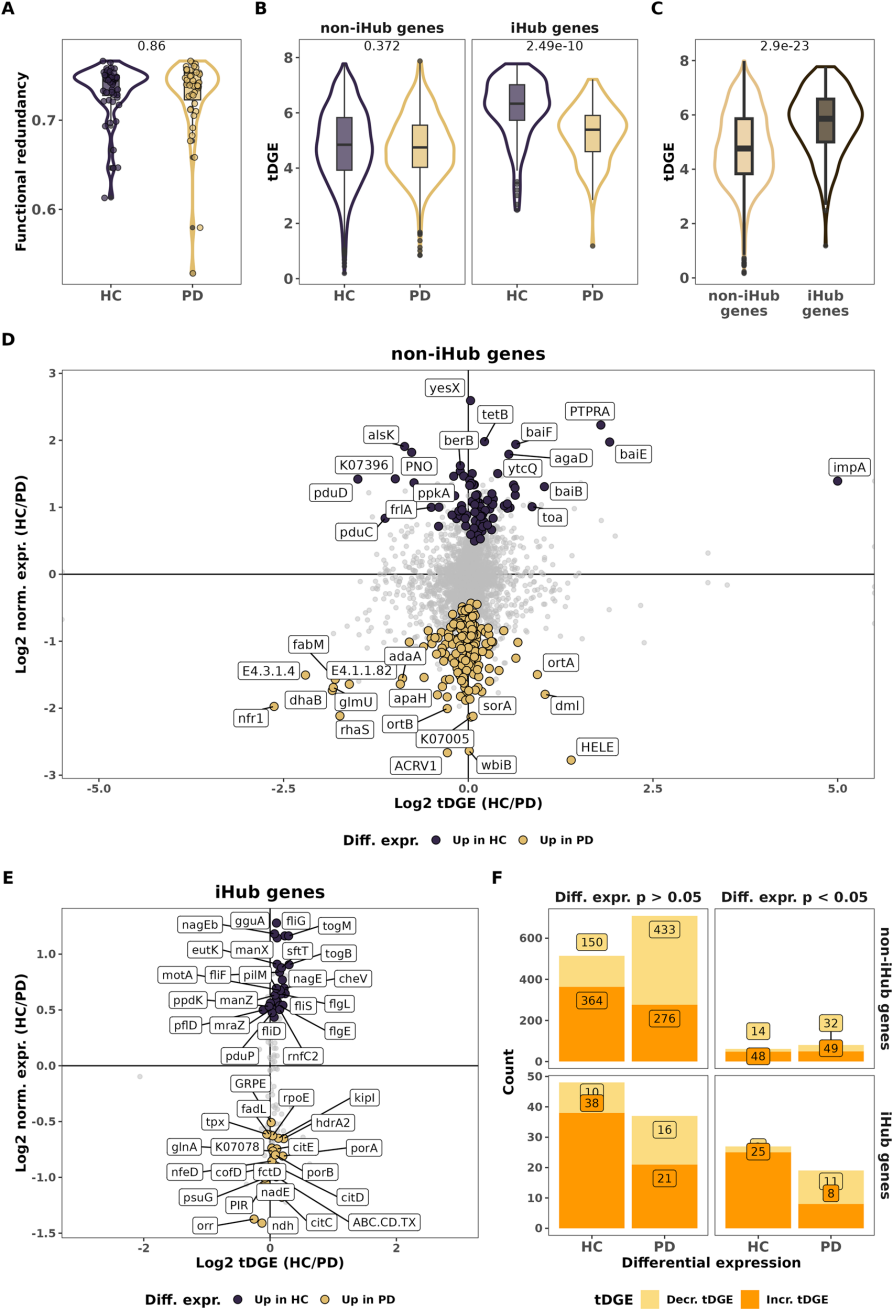
Gene diversity is decreased in PD. **A** Boxplot representing functional redundancy for each sample according to disease status. **B** Boxplot representing gene expression diversity according to disease status. **C** Boxplot representing gene expression diversity grouped by hub genes belonging or not. **A**–**C** figures represent p-values from Mann-Whitney tests. **D**, **E**. Differential abundance versus gene expression diversity for a given gene for non-Hub genes (**D**) and Hub genes (**E**). Y-axis represents log2-fold change of normalized expression and X-axis the log 2-fold change of gene expression diversity. Dots are labelled and coloured for genes with *p* value < 0.05. **F** Stacked bar plot representing the counts of genes with an increased or decreased tDGE for iHub and non-iHub genes. Genes are classified into the PD or HC groups according to the sign of log2FC of normalized expression and faceted according to DEG significance.

After noting interesting modifications in the co-expression network and the lack of hub genes in PD-associated modules, we decided to investigate functional redundancy and the taxonomic diversity of gene expression (tDGE) to assess the impact of taxonomic differences at the level of gene expression. Here we define tDGE as the diversity of taxa expressing a specific gene, while functional redundancy denotes the measure of taxonomic and functional diversity present within a sample (Tian et al., 2020). Our subsequent analyses aimed at differentiating between genes found in module association to either one of the conditions (trait-association, Fig. 6A–C) and genes with differential expression showing an increase or decrease in PD (Fig. 6D–F), all the relevant data are also presented in Supplementary Data 3–7. We did not observe a difference in functional redundancy between HC and PD individuals (Fig. 6A, *p* > 0.05, Wilcoxon test). Interestingly, we found no significant differences for overall tDGE for non-iHub genes but a significantly lower tDGE for iHub genes in PD (Fig. 6B, Wilcoxon test, *p* > 0.05 and *p* < 0.01, respectively). We also noticed iHub genes had a higher tDGE than non-iHub genes (Fig. 6C, Wilcoxon test, *p* < 0.001). We next compared the differential expression and functional diversity of a given gene. We found that gene expression was linked to either an increase in tDGE (more microbes expressing a given gene) or a loss in tDGE (less microbes expressing a given gene) (Fig. 6D, E).

We were able to categorize genes into two groups: those with increased expression linked to a higher tDGE, and those with increased expression linked to a lower tDGE. We finally looked at the proportion of genes within these two above-mentioned categories. We found that a significantly higher proportion of genes with decreases in tDGE in PD compared to HC for both hub genes and non-hub genes that had significantly different expression (*p* = 0.0004 and *p* = 0.049, Fisher exact test and Chi-square test, respectively, Fig. 6F). Of note, we noted the same link between tDGE and differential expression in PD for genes that did not reach significance (Fig. 6F). Interestingly, iHub genes with significantly increased differential expression in PD had a higher fraction of decreased tDGE than non-iHub genes, the difference was however not significant (57% and 43%, respectively, *p* = 0.23, Chi-square test, Fig. 6F).

Finally, we assessed the diversity of functions expressed in the selected taxa, *PeH17* and *Faecousia*. We found a consistent decrease in diversity for the genes expressed by *Roseburia* and *Eubacterium* genera by considering numbers of distinct genes expressed, the inverse Simpson index and the Shannon index (*p* < 0.05, Mann-Whitney test, Supplementary Fig. 5). Additionally, we also found a statistically significant decrease for *Lachnospira* and *Blautia* genera when quantifying the numbers of genes expressed (*p* < 0.05 and *p* < 0.01, Mann-Whitney test, respectively, Supplementary Fig. 5). We also noted an increase in the inverse Simpson index for *Faecousia* and in the number of genes expressed by *PeH17*. However, these increases did not reach statistical significance (*p* = 0.084 and *p* = 0.082, respectively, Mann-Whitney test, Supplementary Fig. 5).

## Discussion

Most knowledge on microbiome perturbations and associations with diseases comes from either 16S rRNA gene or whole genome sequencing which do not allow the ability to assess transcriptional activity of the microbiome. We previously demonstrated significant alterations of the gut microbiome in individuals with PD and highlighted strong differences in the expression of flagellar assembly and chemotaxis genes^19^. Here, we aim at analysing the dataset using a system ecology approach to uncover microbial key regulator functions associated with PD. More specifically, we employ a co-expression network-based approach on MT and MG data to assess the microbiome-wide impacts of these changes. We identify strong associations between co-expression network modules and disease status, with four modules linked to HC and five to PD. Additionally, we find eight modules that were not associated with either HC or PD. Except for module M1, that is mainly a module comprised of genes not clustering with other genes, we find higher values of degree and closeness centrality for non-associated modules compared to the trait-associated modules. Given their overall high centrality and the lack of trait association, these modules may represent stable, core functions that support the integrity of microbial ecosystem services in both health and disease. We found interesting that module diversity was anti-correlated with eigenvector and betweenness centrality, as if diverse modules have less short path and less strong interactions with their relative co-clustered genes. To our knowledge, there is no paper showing these correlations/anti-correlations between the module gene diversity and centrality measures, this will need to be reproduced and re-assessed to understand the full biological meaning of this finding. Overall, this work will need to be reproduced on PD or other pathologies, to distinguish core and stable functions from the labile and adaptive functions in the gut microbiome.

Hub genes represent biologically relevant properties and are more likely to reflect key functions that might be lost in diseased individuals such as PD^25–28^. Therefore, identifying hub genes is a valuable strategy to describe key functions that define healthy versus diseased individuals. Using this approach, we first select the 95th percentile of connected genes from modules associated with either HC or PD to uncover the most important genes in the co-expression network. This strategy reveals that 95% of the hub genes are associated with HC. To gain more insight into the PD-associated microbiome, we select the top 10% most connected genes per module, defined here as iHub genes. With this approach, we uncover citrate lyase as being enriched in PD. We can link the taxonomic expression of these genes to *PeH17* spp. and *Faecousia* spp. Interestingly, *PeH17* along with *Christensenella* are part of the *Christensenellales* order, the latter is usually described as increased in PD^29,30^ and cross-feeding with *Methanobrevibacter*^31^. Of note, *PeH17* is poorly characterised, and we currently do not know if *PeH17* exhibits the same cross-feeding activities with *Methanobrevibacter*. To our knowledge, there is no record of citrate lyase genes being associated with disease in humans and so far, no evidence of *PeH17* being associated with a disease in humans. Interestingly, thirteen genes from the flagellar assembly pathway are iHub genes and nine when using the 95^th^ percentile approach, showing once more the importance of this pathway in the microbial network, especially in the context of PD. Finally, the high number of hub genes from HC-associated modules is noteworthy, as it indicates that there is clear shift in the expression of key functions in the gut microbiome of PD.

Amongst modules, we find that M2 (HC-associated module) is of particular interest based on its connectivity, module diversity and centrality. M2 comprises the most connected genes and especially genes from the pdu and eut operons, two operons involved in the assembly of bacterial microcompartments. These operons are responsible for the utilization of ethanolamine and 1,2-propanediol, an important energy source for bacteria and typically associated with pathogenic bacteria^32,33^, as they confer a growth advantage by utilizing abundantly present 1,2-propanediol and ethanolamine (EA)^34,35^. However, it has been recently described that a wide range of commensals are also expressing these genes^36–39^. We find that the expression of these genes is decreased in PD compared to HC, especially in genera such as *Blautia* and *Anaerobutyricum* but increased in *Flavonifractor plautii*, a bacterial species that we previously highlighted as associated with HC^19^. In addition to BMC genes, we find genes from the togBMNA operon as hub genes and with genes significantly decreased in PD. These genes are part of ABC transporters involved in pectin degradation and production of short chain fatty acids (SCFAs)^40^. We also note the presence of lacFG, ganPQ, wzm/wzt (M2) and other members of ABC transporters responsible of polysaccharides/polyols transport. Resolving the corresponding taxa reveals that these genes are significantly downregulated in members of *Faecalibacterium*, *Gemmiger*, *Roseburia* and *Blautia* genera. Along with the decrease in expression of BMC genes in PD, important metabolic processes, such as polyols transport (galacturonides, rhamnose, lactulose, etc.), are clearly downregulated in PD gut microbiome. Gut commensals lose keystone functions in the context of PD, resulting in the apparent loss of a gut microbial functional equilibrium which should explain the taxonomical shifts noted previously.

In addition to the functional shifts seen in PD, we highlight strong correlations between the expression of BMC and FA genes even though these genes are not expressed by the same taxa. Indeed, BMC genes are mainly expressed by *Blautia, Anaerobutyricum* and *Faecalibacterium* species while FA are mainly expressed by *Roseburia, Lachnospira* and *Eubacterium* species. Interestingly, a recent study reported that GH26 b-1,4-endomannanase, a glycosyl hydrolase present on the surface of *R. intestinalis* but absent in *F. prausnitzii*, was necessary to grow on gluco-galactomannans^41^. Our observation of reduced expression levels of BMC genes and polyol transporters in genera such as *Blautia*, *Anaerobutyricum*, and *Faecalibacterium*, along with the downregulation of FA, suggests a decline in cross-feeding interactions amongst these commensals in the gut microbiome of PD. However, it remains unclear whether the decreased expression of FA precedes or follows the reduced expression of BMC genes and polyol transporters. Interestingly, *Faecalibacterium*, *Blautia* and *Roseburia* species abundances are also decreased in other diseases such as IBD^42^, cancer^43–46^ or Type 2 diabetes^47^, underpinning the importance of these specific taxa.

We also observe a decreased tDGE in PD gut microbiome but no differences in overall functional redundancy. However, and crucially, we show here that most of the genes overexpressed in PD are linked to a decrease in the diversity of taxa expressing these functions. Interestingly, this decrease was even more apparent for genes that we defined as iHub genes, highlighting the reduced expression of keystone genes, such as BMC genes, in PD linked to keystone taxa of the gut microbiome, most notably *Blautia* species. In contrast to PD, in HC-associated genes, we note an opposite relationship, whereby an increased gene expression is linked to increased tDGE. Moreover, we found a decrease in functionality at the genus level. Indeed, *Roseburia*, *Blautia* and *Eubacterium* are expressing fewer genes in PD. On the other hand, we show here that *Faecousia* and *PeH17*, even if not reaching statistical significance, exhibit an increased diversity in genes expressed in the PD gut microbiome. All in all, this is to our knowledge, the first account of a decreased diversity in transcriptomic expression and functionality in the PD gut microbiome, bringing a new functional and ecological understanding to the concept of human gut microbiome dysbiosis.

Using the present study design, we were unable to measure resilience and stability of the gut microbiome in PD in comparison to healthy individuals as these measures are time-dependent. Nonetheless, a general decrease in taxa expressing key functions should result in, or be secondary to, decreased resilience and stability, as previously hypothesised^48,49^. Future studies should aim at characterising the gut microbiome in a longitudinal manner to evaluate the dynamics of the gut microbiome in a disease context. It is important to note that PD is a late onset and long-lasting progressive disease, understanding early perturbations in the gut ecosystem would enable assessment of the association between the gut microbiome and PD progression.

So far, little is known about the dynamics of the gut microbiome and the possibility of recovering from a dysbiotic gut microbiome in PD and during PD progression. In the same line, the dynamics and resilience of faecal transplantations, an emerging therapeutical option to recover from microbiome dysbiosis, are poorly understood. Human to mouse or human to organ-on-chip faecal transplantations studies are required to test the possibility to recover keystone functions from a dysbiotic gut microbiome.

In conclusion, we report here the importance of studying the functional capacity and diversity in the gut microbiome of individuals with PD. Furthermore, we want to highlight the significance of complementary approaches in understanding the microbiome, which is both fascinating and complex in its diversity and versatility. While differential abundance analysis and multiple regression analysis are valuable, they may fail to appreciate the intricate web of interactions by not accounting for gene co-expression and interdependencies. We see here, an overall decreased taxonomic diversity of gene expression and a decreased of functional diversity for keystone taxa. We also highlight de dysregulation of the cross-feeding of the gut microbiome in health and disease. *Faecalibacterium*, *Blautia* and *Roseburia* species are distinguishing themselves as keystone species in the healthy gut microbiome. Efforts are required to deepen our understanding of the interdependencies among these species, as well as to investigate ways to stimulate the functions of these taxa. Ultimately, in alignment with the ecological principles, researchers and clinicians should focus on rewilding the gut microbiome’s functions in diseased individuals to restore the diversity of functions within the gut microbiome ecosystem.

## Methods

### Study population

All subjects from both cohorts provided informed written consent, and the sample analysis was approved by the Comité National d’Ethique de Recherche of Luxembourg (reference no.: 140174_ND). The DeNoPa cohort represents a prospective, biannual follow-up study of (initially de novo*)* Parkinson’s disease (PD) patients at the Paracelsus-Elena Klinik, Kassel, Germany. Fecal samples from PD patients (46) and healthy controls (29) were collected during the 4-year follow-up visit for the cohort. Details on inclusion and exclusion criteria and ancillary investigations have been published previously^50,51^. DeNoPa subjects were required to have a 4-week antibiotic free interval before fecal sample collection. As additional control subjects, we collected fecal samples from (20) neurologically healthy subjects living in the same household as the DeNoPa participants. Samples of de novo PD patients from a cross-sectional cohort at the same clinic were included if subjects were recently diagnosed, drug-naïve and met United Kingdom Parkinson’s Disease Society Brain Bank (UKPDSBB) clinical diagnostic criteria^52^. All subjects except household HC were interviewed and examined by an expert in movement disorders. The study conformed to the Declaration of Helsinki and was approved by the ethics committee of the Physician’s Board Hessen, Germany (FF 89/2008). The DeNoPa trial is registered at the German Register for Clinical trials (DRKS00000540).

### Sample preparation

Fecal samples were collected at the clinics via a stool specimen collector (MedAuxil) and collection tubes (Sarstedt), as previously described^53^. Samples were immediately flash-frozen on dry ice after collection. Samples were subsequently stored at –80 °C and shipped on dry ice. Extractions from fecal samples were performed according to a previously published protocol^54^ conducted on a customized robotic system (Tecan Freedom EVO 200). After extraction, DNA and RNA were purified prior the sequencing analysis by using the following commercial kits respectively: Zymo DNA Clean&Concentrator-5 (D4014) and Zymo RNA Clean&Concentrator-5 (R1014). RNA quality was assessed and quantified with an Agilent 2100 Bioanalyser (Agilent Technologies) and the Agilent RNA 6000 Nano kit, and genomic DNA and RNA fractions with a NanoDrop Spectrophotometer 1000 (Thermo Scientific) as well as commercial kits from Qubit (Qubit ds DNA BR Assay kit, Q32850; Qubit RNA BR Assay kit, Q10210). All DNA samples were subjected to random shotgun sequencing. Following DNA isolation, 200–300 ng of DNA was sheared using a Bioruptor NGS (Diagenode) with 30 s ON and 30 s OFF for 20 cycles. Sequencing libraries were prepared using the TruSeq Nano DNA library preparation kit (Illumina) following the manufacturer’s protocol, with 350 bp average insert size. For MT, 1 µg of isolated RNA was rRNA-depleted using the RiboZero kit (Illumina, MRZB12424). Library preparation was performed using the TruSeq Stranded mRNA library preparation kit (Illumina) following the manufacturer’s protocol, apart from omitting the initial steps for mRNA pull down. MG and MT analyses, the qualities of the libraries were checked using a Bioanalyzer (Agilent) and quantified using Qubit (Invitrogen). Libraries were sequenced on an Illumina NextSeq500 instrument with 2 × 150 bp read length. We retrieved an average of 50 and 25 million reads per sample for MG and MT respectively.

### Metagenomics and metatranscriptomics generation

For all samples, MG and MT sequencing data were processed and hybrid-assembled using the Integrated Meta-omic Pipeline (IMP)^55^. Data was quality trimmed, adapter sequences were removed, MT rRNA reads were removed by mapping against SILVA 138.1 (RRID:SCR_006423)^56^ and human reads were removed from MT and MG after mapping against the human genome (hg38) and transcriptome (RefSeq 212, RRID:SCR_003496). Pre-processed MG and MT reads were co-assembled using the IMP-based iterative hybrid-assembly pipeline using MEGAHIT (1.0.3, RRID:SCR_018551)^57^, for more information on co-assembly please consult the original publication^55^. After assembly, the prediction and annotation of genomic features such as open-reading frames (ORFs) and non-coding genes was performed using a modified version of Prokka (version 1.14.6, RRID:SCR_014732)^58^ and followed by functional annotation of those using Mantis (RRID:SCR_021001)^59^. We modified the Prokka Perl script to disable the Prodigal gene prediction call to obtain also incomplete ORFs, plus disable the time-consuming protein annotation sub-workflow of Prokka. Genomic features were quantified on MG and MT level using featureCounts (RRID:SCR_012919)^60^ from the final gff file. Taxonomic annotation of reads and contigs was performed using Kraken2 (RRID:SCR_005484)^61^ with a GTDB release207 database (RRID:SCR_019136) (http://ftp.tue.mpg.de/ebio/projects/struo2/GTDB_release207/kraken2) and a 0.75 confidence threshold.

### Co-expression network construction

Prior to network construction, MG and MT counts were normalized to transcripts per million (TPM). This choice was motivated by prior usage of this normalizing method and the validation of this approach done by Cho et al.^62^. For this purpose, we used the featureCounts output. Briefly, we accounted for both sequencing depth and gene length, as is standard for RNAsequencing data. TPM values were computed as: $\frac{{R}_{i}/{L}_{i}}{\mathop{\sum }\limits_{j=1}^{N}{R}_{j}/{L}_{j}}\times {10}^{6}$, where $R$ is read count for gene $i$, $L$ is gene length for gene $i$, $N$ is the total number of genes.

We created normalised gene expression by using a ratio of MT TPM to MG TPM as described before^22^. We then used the Python implementation of WGCNA (RRID:SCR_003302) (PyWGCNA, version 2.0.4) to construct a signed co-expression network of KEGG orthologs (KO) normalised gene expression from the microbiome of individuals with PD and HC according to the WGCNA procedure^63^. Normalised gene expression of KOs was power transformed prior to WGCNA, using PowerTransformer from *sklearn.preprocessing* (RRID:SCR_019053). The *WGCNA* function was run with the following parameters: minimum module size minModuleSize = 20, dissimilarity threshold MEDissThres = 0.18, networkType = ’signed’. Modules were identified using average linkage hierarchical clustering and the dynamic tree-cut function. The power parameter was set to 17, the lowest value achieving a scale-free topology criterion (SFT.R.sq ≥ 0.9; power =; 17: SFT.R.sq = 0.918) while retaining biologically relevant connectivity (mean(k) = 1.50, k-connectivity).

### Topology metrics calculation

To analyse the structure of each network module, we calculated several key topological metrics. Module size was defined by the number of genes (nodes) in each module. Intramodular connectivity was measured as the sum of edge weights in the module adjacency matrix, divided by two. Mean connectivity represented the average edge weight within the module. Module diversity was assessed using the Shannon index, calculated on the sum of MT/MG ratio of genes in a module (see ‘Diversity measures’ for more detail). Centrality and clustering metrics were calculated using various functions from NetworkX (nx) (https://networkx.org, RRID:SCR_016864)^64^, python package for the construction and analysis of complex networks. Specifically, clustering coefficient was calculated with *nx.average_clustering*, closeness centrality with *nx.closeness_centrality*, degree centrality with *nx.degree_centrality*, eigenvector centrality with *nx.eigenvector_centrality_numpy*, and betweenness centrality with *nx.betweenness_centrality*.

### Hub genes and intramodular hub genes selection

Hub genes are defined as the top 5% most connected genes present in trait-associated modules. For this purpose we used the 95th percentile of connectivity for all modules with a significant trait-association using the *quantile()* function from R base package (RRID:SCR_001905). We also defined intramodular hub genes (iHub genes) by selecting 10% of the most connected genes from trait-associated modules.

### Selection of taxa of interest

In order to support the potential links between BMC and FA gene expression, we selected taxa of interest based on literature evidence of either flagellin expression, BMC formation and significant expression of either FA or BMC genes. In that manner we selected the taxa belonging to the following genera: *Agathobacter, Anaerobutyricum, Blautia, Faecalibacterium, Flavonifractor, Eubacterium, Lachnospira, Roseburia, RUG115* and all genera from *Lachnospiraceae*, *Oscillospiraceae* and *Ruminococcaceae* comprising ‘CAG’ in their genus name.

### Diversity measures

We defined module diversity by the richness and evenness of normalised gene expression within a given module, whereby we summed normalised expression for each gene and applied the Shannon index from the vegan R package to the summed normalised expression (2.6.6.1, RRID:SCR_011950)^65^. Hence, the module diversity index is calculated as following: ${{mH}}^{{\prime} }=-\sum gi\,{{\text{log}}}_{b}\,gi$, where ${mH}{\prime}$ is the module diversity index and ${gi}$ is the normalised expression of gene $i$.

We defined tDGE as the diversity of taxa expressing a given gene using the Shannon index. Hence, the tDGE index is calculated as following: ${{gH}}^{{\prime} }=-\sum si\,{{\text{log}}}_{b}\,si$, where ${{gH}}^{{\prime} }$ is the tDGE index for gene $g$ and ${si}$ is the normalised expression of species $i$ expressing the gene $g$.

Functional redundancy was calculated using the R package SYNCSA (1.3.4) using the function *rao.diversity()*^66^, functional redundancy is thereby defined as the difference between species diversity and the functional diversity (measured by Rao quadratic entropy) as proposed previously (de Bello et al. 2007).

We quantified the diversity of gene expressed per taxa by summing the normalised expression, for each sample, at the genus level for the selected taxa (see above), *PeH17* and *Faecousia*. For that purpose, we used the observed number of genes expressed, the inverse Simpson index (vegan package) and Shannon index.

### Statistics and plotting

All statistics and plots were handled in R (4.4.1) using Rstudio (2024.4.2.764, RRID:SCR_000432). Wilcoxon signed-rank tests, spearman correlations tests and FDR corrections were performed using *rstatix* package (0.7.2, RRID:SCR_021240). Gene set enrichment analysis was conducted using fgsea R package (1.30.0, RRID:SCR_020938). Differential expression analysis was performed using DeSeq2 R package (1.42.1, RRID:SCR_015687). We refer to p- and q- value in the text to depict features that were significant before FDR correction (*p* < 0.05) and features remaining significant after FDR correction (*q* < 0.05). All plots were produced using ggplot2 (3.5.1, RRID:SCR_014601) and R graphics functions.

## Supplementary information

Supplemental Figures

Supplementary data

## Data Availability

Metagenomic and metatranscriptomic data are available at on SRA, under the BioProject: PRJNA782492.

## Code availability

The IMP pipeline, which was used for analysis of metagenomic and metatranscriptomic data, is available at https://gitlab.lcsb.uni.lu/IMP/imp3. The R and python code used for statistical analyses and visualizations is available at https://gitlab.com/uniluxembourg/lcsb/systems-ecology/co_expression_network.
