# Supplementary material for: Human gut microbiome gene co-expression network reveals a loss in taxonomic and functional diversity in Parkinson’s disease
Source: NPJ Biofilms Microbiomes. 2025 Jul 24;11:142. doi: 10.1038/s41522-025-00780-0 (PMC12289924; doi:10.1038/s41522-025-00780-0)

Supplementary Figure 1. Gene and species prevalence using MTA as a proxy of microbial activity

A. Count of gene that are shared by 50% of the individuals, label depicts the percentage instead. **B** and **C.** Gene and species prevalence represented as dot plot proportional to the number of a given feature. **D.** Prevalence of genes reported to their respective average TPM value, fold change, median value and variance between groups.

Supplementary Figure 2. Differential MTA at the genus and species level.

A. Volcano plot of the genus differential MTA. **B.** Volcano plot of the genus differential MTA. Dots are colorized according to the fold change direction, shaped according to the significance and sized according to the mean MTA found in HC group. Differential abundance analysis was performed using a Wilcoxon signed rank test.

Supplementary Figure 3. Additional topology metrics from WGCNA.

A. Boxplots representing betweenness, closeness and eigenvector centrality. Kruskal and Wallis test. **B.** Correlation between topology metrics and module diversity. All tests are based on the Spearman correlation. **C.** Heatmap representing the correlation between various topology metrics. Tests are Spearman correlation test.

Supplementary Figure 4. KEGG pathways per module.

Counts of KEGG pathways per module for the hub genes. The size of the dots represents the proportion of a given pathway within a module.

Supplementary Figure 5. BMC metabolism and ABC transporter gene expression resolved by taxa.

A. Normalised gene expression for BMC metabolism, genes are grouped based on the described enzymatic activities. **B.** Normalised expression for togBMN genes for bacterial species with significant differential expression. **C.** Normalised expression for additional ABC

transporter genes found as hug genes, for bacterial species with significant differential expression. All tests are Mann-Whitney tests corrected with FDR.

Supplementary Figure 6. BMC genes correlate with flagella assembly genes.

Heatmaps representing spearman correlation coefficients between genes involved in BMC formation, catabolism or anabolism and genes involved in flagellar assembly. **A.** Heatmap correlation tests for selected bacteria using normalized expression. **B.** Heatmap correlation tests for selected bacteria using MT TPM.

Supplementary Figure 7. Diversity of genes expressed by *Roseburia*, *Blautia* and *Eubacterium* genera is decreased in PD, but increased for *Faecousia.*

Alpha diversity measures of gene expressed in each genus. Plot are representing the sum of normalised expression for each sample and for each selected genus (see Material and Methods), with the addition of the *PeH17* and *Faecousia* genera. Values are representing the different diversity measures: inverse Simpson index, observed number of genes and Shannon index. All p-values are from a Mann-Whitney test.

Supplementary Figure 1

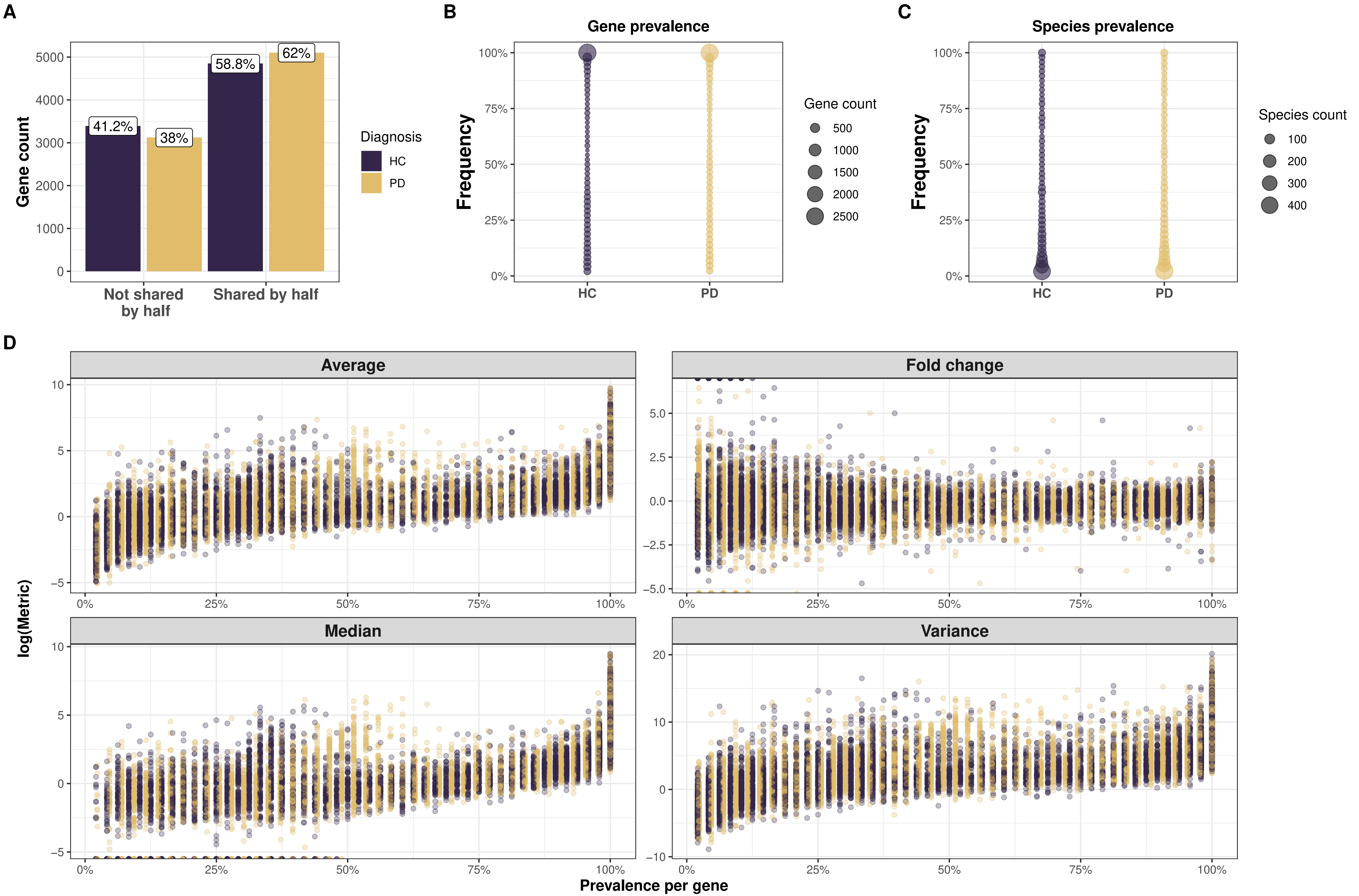

Supplementary Figure 2

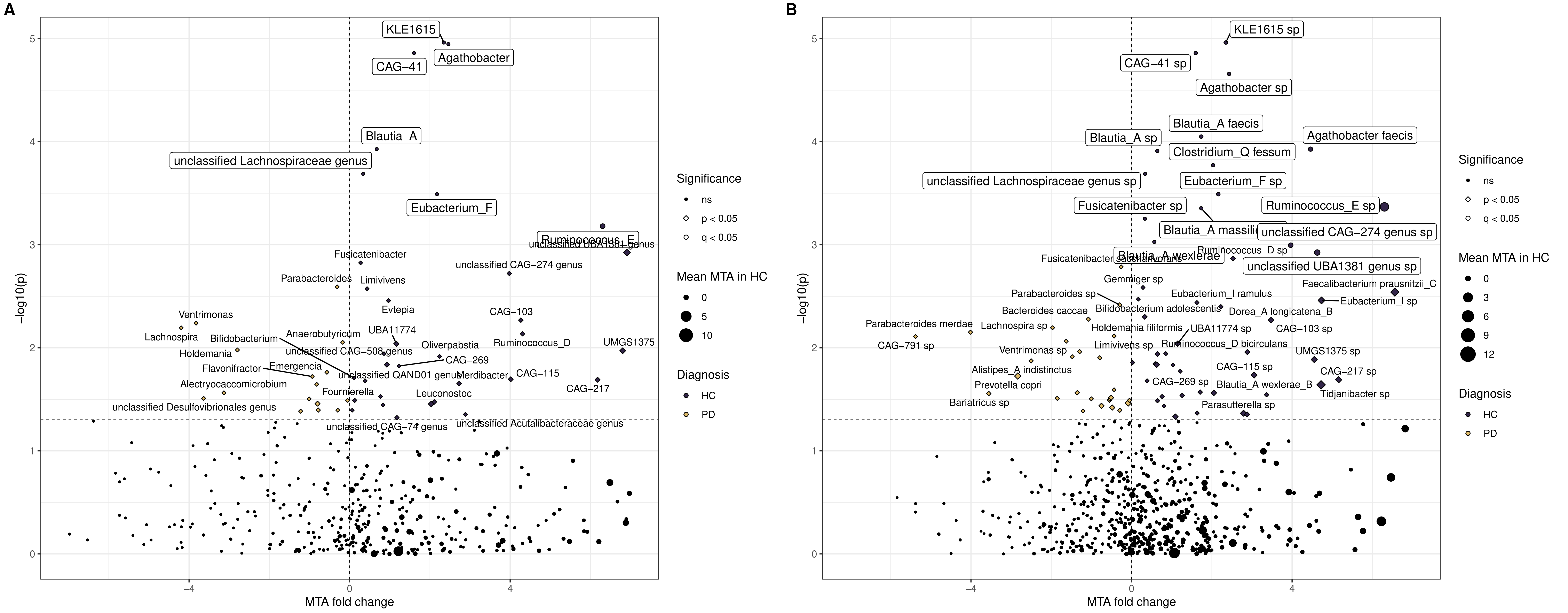

Supplementary Figure 3

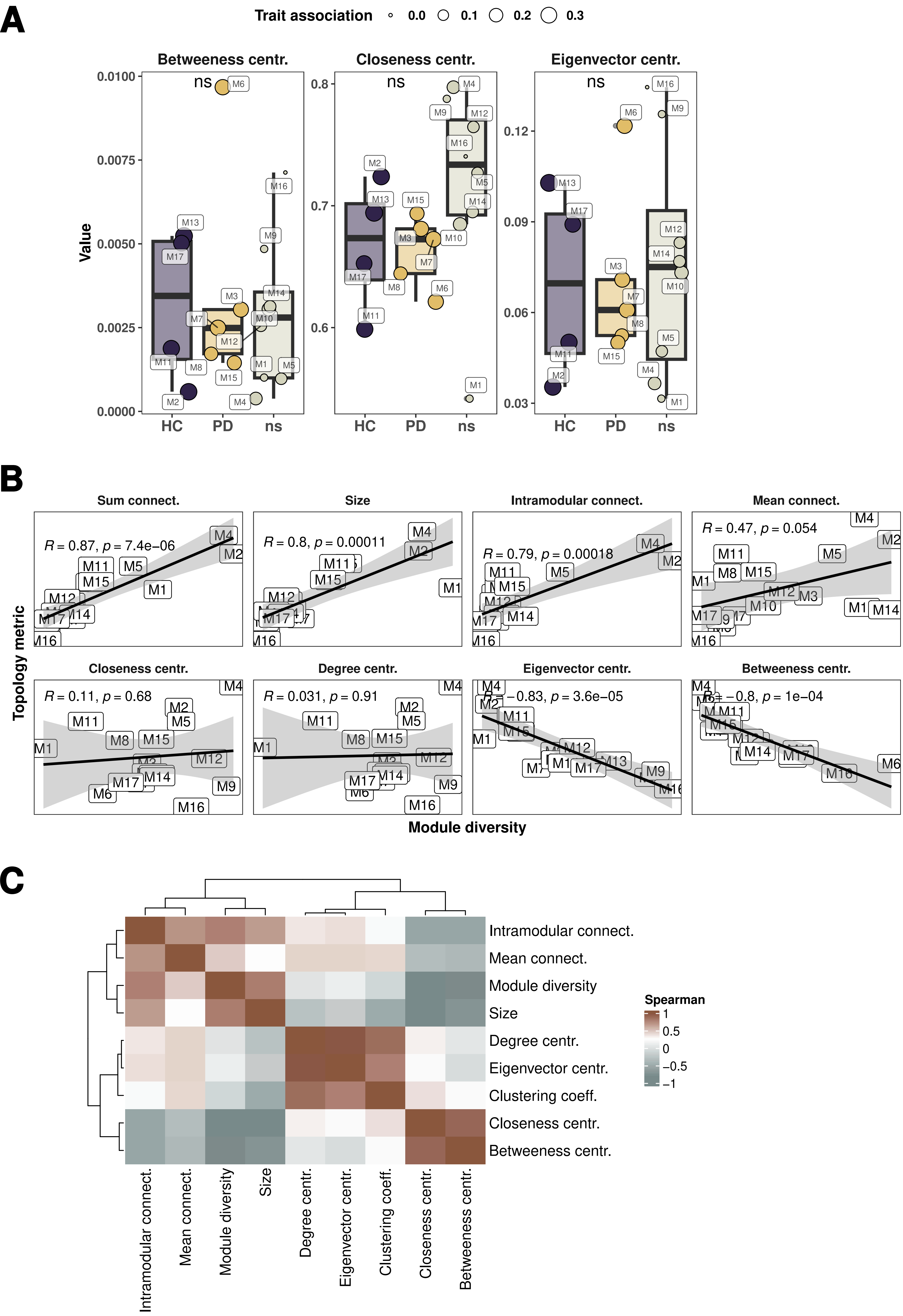

Supplementary Figure 4

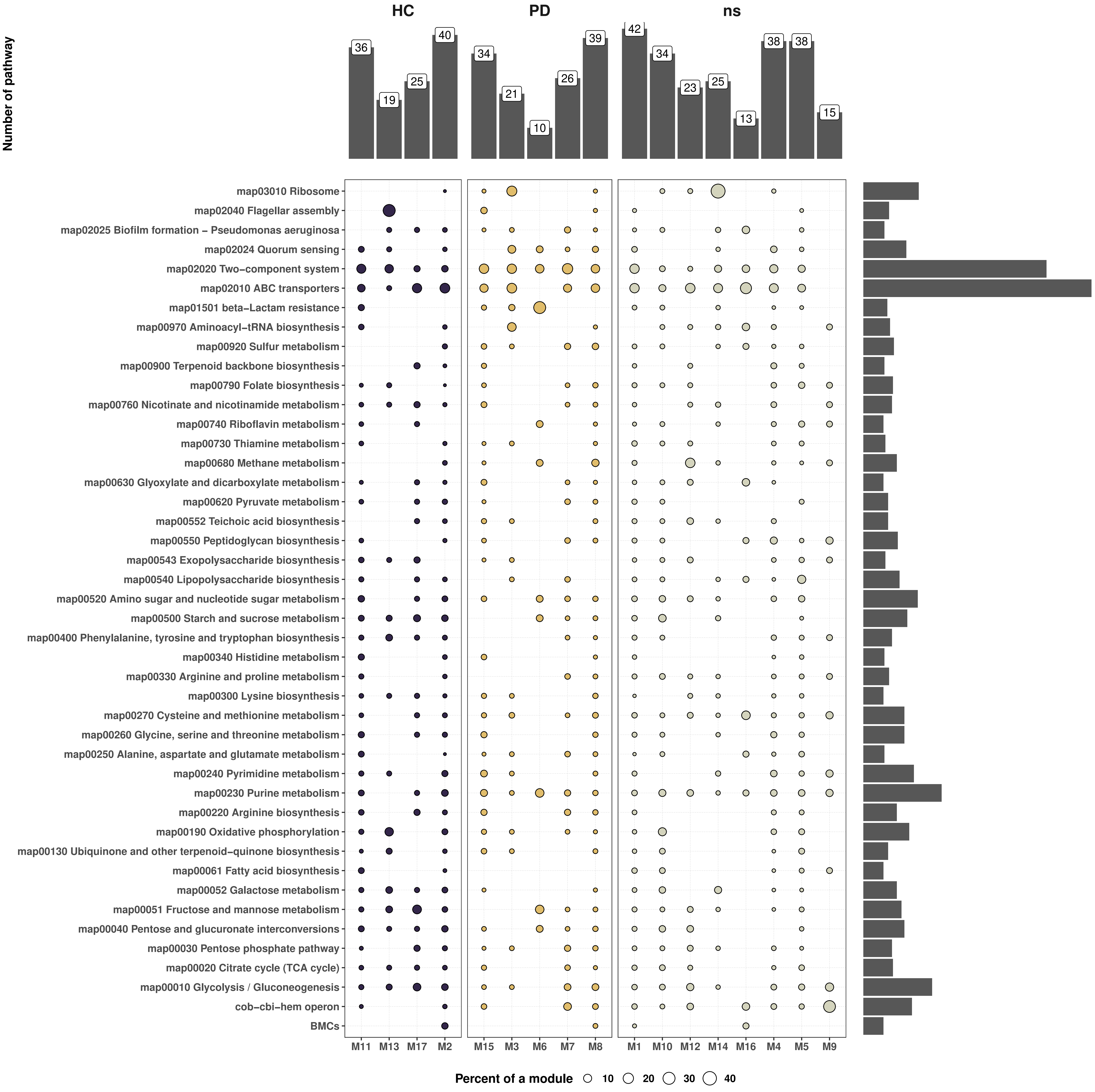

Supplementary Figure 5

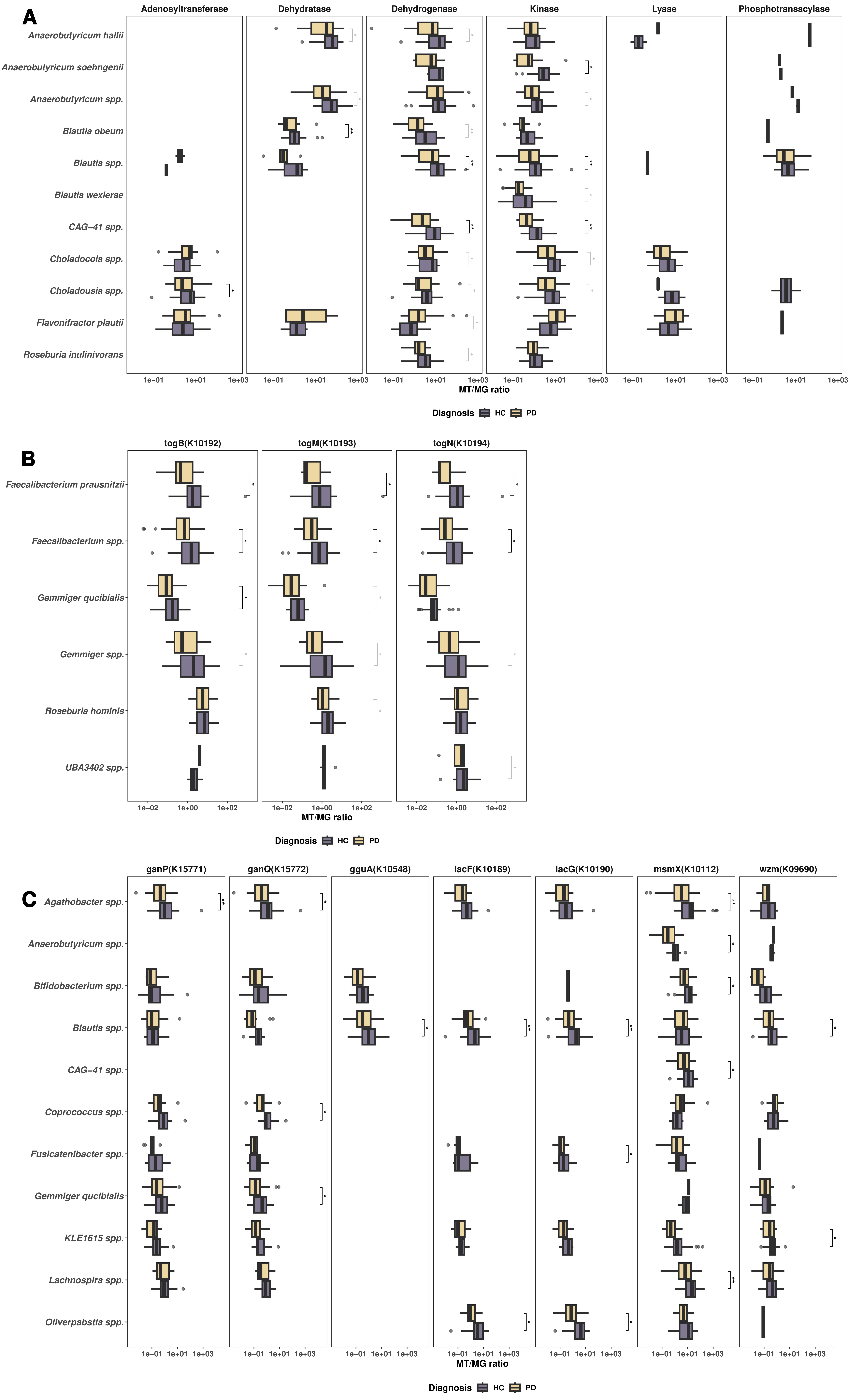

Supplementary Figure 6

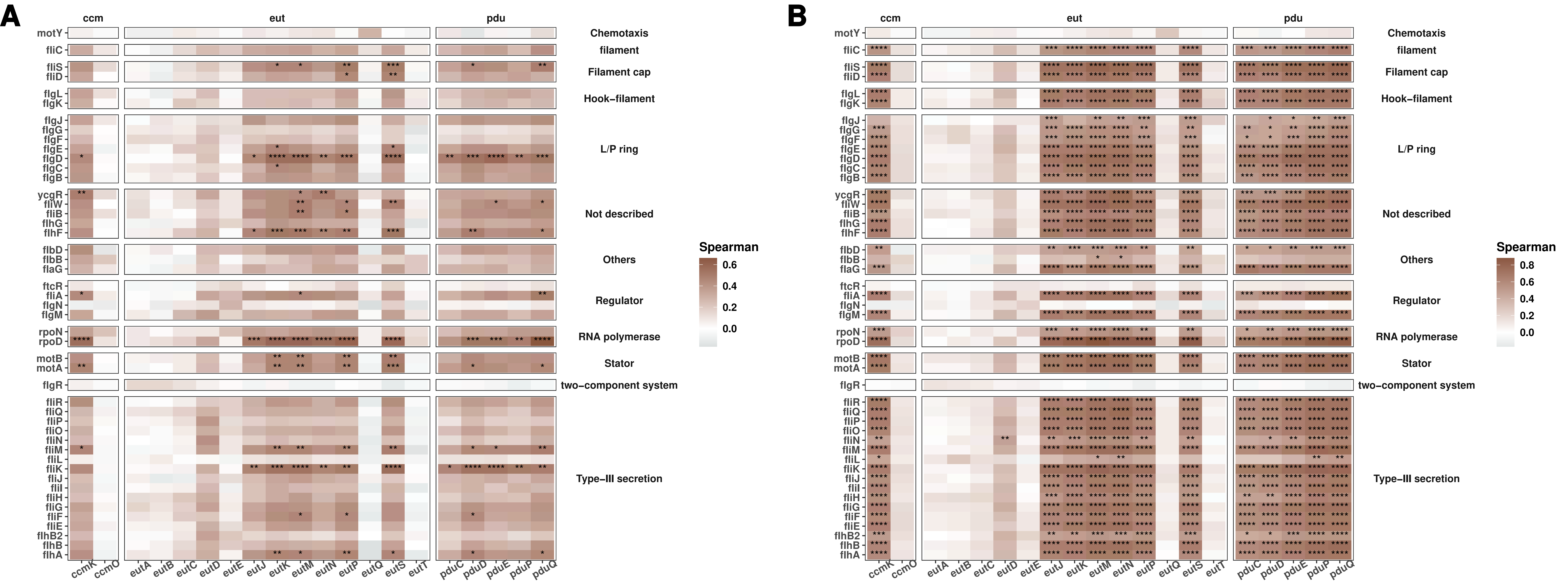

Supplementary Figure 7

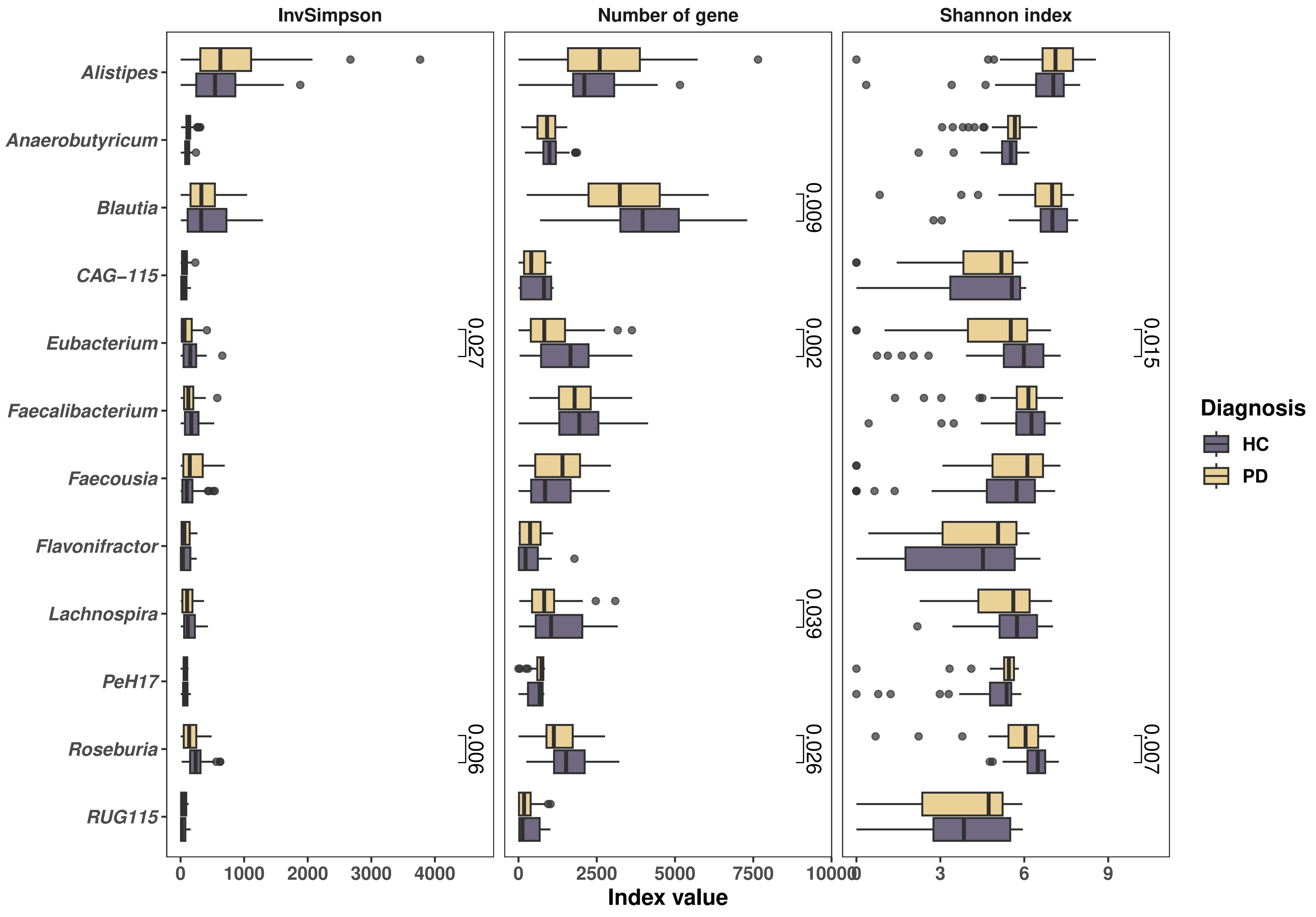

Supplement: Supplementary file 1 — Supplemental Figures [file 41522_2025_780_MOESM1_ESM.pdf]
